# Perceptions of Educational Needs in an Era of Shifting Mental Health Care to Primary Care: Exploratory Pilot Study

**DOI:** 10.2196/32422

**Published:** 2022-01-07

**Authors:** Stephanie Sutherland, Dahn Jeong, Michael Cheng, Mireille St-Jean, Alireza Jalali

**Affiliations:** 1 Department of Critical Care, The Ottawa Hospital Ottawa, ON Canada; 2 Department of Innovation in Medical Education Faculty of Medicine University of Ottawa Ottawa, ON Canada; 3 Department of Psychiatry Faculty of Medicine University of Ottawa Ottawa, ON Canada; 4 Children's Hospital of Eastern Ontario Ottawa, ON Canada; 5 Department of Family Medicine Faculty of Medicine, University of Ottawa Ottawa, ON Canada

**Keywords:** mental health, Canada, qualitative research, caregiver, family physician, mentorship

## Abstract

**Background:**

There is an unmet need for mental health care in Canada. Primary care providers such as general practitioners and family physicians are the essential part of mental health care services; however, mental health is often underestimated and underprioritized by family physicians. It is currently not known what is required to increase care providers’ willingness, comfort, and skills to adequately provide care to patients who present with mental health issues.

**Objective:**

The aim of this study was to understand the need of caregivers (family members overseeing care of an individual with a mental health diagnosis) and family physicians regarding the care and medical management of individuals with mental health conditions.

**Methods:**

A needs assessment was designed to understand the educational needs of caregivers and family physicians regarding the provision of mental health care, specifically to seek advice on the format and delivery mode for an educational curriculum to be accessed by both stakeholder groups. Exploratory qualitative interviews were conducted, and data were collected and analyzed iteratively until thematic saturation was achieved.

**Results:**

Caregivers of individuals with mental health conditions (n=24) and family physicians (n=10) were interviewed. Both the caregivers and the family physicians expressed dissatisfaction with the status quo regarding the provision of mental health care at the family physician’s office. They stated that there was a need for more educational materials as well as additional support. The caregivers expressed a general lack of confidence in family physicians to manage their son’s or daughter’s mental health condition, while family physicians sought more networking opportunities to improve and facilitate the provision of mental health care.

**Conclusions:**

Robust qualitative studies are necessary to identify the educational and medical management needs of caregivers and family physicians. Understanding each other’s perspectives is an essential first step to collaboratively designing, implementing, and subsequently evaluating community-based mental health care. Fortunately, there are initiatives underway to address these need areas (eg, websites such as the eMentalHealth, as well as the mentorship and collaborative care network), and information from this study can help inform the gaps in those existing initiatives.

## Introduction

Family physicians are most often the first point of contact for patients presenting with mental health illnesses. Unfortunately, mental health is often underestimated and underprioritized by family physicians [1,2]. In total, 75% of mental health visits are related to mood and anxiety disorders, and the majority of these mental health visits occur in the primary care setting [3,4]. Family physicians are central to address mental health illnesses in their communities as they often have the advantage of a previously established relationship, ease of access, and can be seen in much less time than a wait to see a psychiatrist [5]. To date, it is unclear what is needed to increase family physicians’ willingness, comfort, and skills to provide care to this complex patient population. Furthermore, it is not known what needs must be addressed to ensure successful and clinically effective transitions in care for patients with schizophrenia to be treated in primary care settings. The literature on health care interventions is clear in that education alone is not a solution to service care provision. Collaborative care models have been shown to improve access to mental health care, individual and population outcomes, and cost-effective care [6]. Yet, there is a dearth of literature to guide educational interventions geared toward the management of schizophrenia in primary care.

Qualitative needs assessments can unlock potential solutions to building capacity within the primary setting for the assessment, treatment, and management of mental health conditions. The objective of this needs assessment was to better understand the education and information needs of (1) caregivers of patients with mental health needs in primary care, with a focus on early recognition, diagnosis, and treatment of schizophrenia, bipolar mood disorder, and depression; and (2) family physicians’ perceptions of barriers to care.

## Methods

### Design

A needs assessment is a systematic process to collect and analyze information on a target group’s needs or “gaps” between current and desired situations. Performing a needs assessment is well accepted as an essential first step in the educational process [7]. Calls for innovative strategies in needs assessment methodology have been made in the medical literature over an extended period. A social constructivist approach focuses our study design to permit for collaborative dialogue to promote understanding and learning among and between stakeholder groups [8]. The current needs assessment employed a qualitative approach to capture the experiences and rich details provided by the 2 stakeholder groups, caregivers and family physicians.

### Sampling and Procedure

The participants were selected through a purposive and snowball sample strategy. Two focus groups with patient caregivers were created. In total, 24 caregivers took part in each of the 2 focus groups. The gender composition of the groups was predominately female with only 1 male participant. All of the women in the focus groups were mothers of adolescent or adult children with mental health conditions and ranged in age from 38 to 72 years. The male in the second focus group was a caregiver for his brother-in-law. Each of the focus groups lasted for 2 hours and followed a semistructured focus group protocol. The focus group protocol was developed from a review of the relevant literature and from expert input from team members MC (a clinical psychiatrist) and MSJ (a family physician). Further, interview questions were vetted with the Canadian Schizophrenic Society to ensure applicability and clarity for use with caregivers. Family physicians affiliated with the Faculty of Medicine at the University of Ottawa were invited to participate in a semistructured interview. In turn, the agreeing participants nominated colleagues who might be willing to participate in an interview. The interview guide was developed from a review of the relevant literature and from expert input from several family physicians associated with the University of Ottawa’s Faculty of Medicine. A total of 10 family physicians took part in a 30-minute interview. The clinical experience level of family physicians ranged from 1 year to 24 years.

### Data Analysis

Data collection and analysis were an iterative process and continued until no new themes arose. In qualitative studies, data saturation occurs when the researchers are no longer obtaining new information or themes. Interviews were audio recorded and transcribed verbatim. Qualitative data analysis techniques were consistently applied to the focus group and interview data. This analysis included 2 of the research team members (SS and AJ) who participated in all coding meetings and the application of inductive coding techniques. Themes were generated directly from the data sets.

### Ethical Considerations

Ethics approval was obtained from the University of Ottawa’s Research Ethics Board.

### Study Rigor

To promote study rigor, all transcripts were sent back to the participants for review and face validation. Two forms of triangulation were employed to achieve a balanced perspective and enhance the reliability of the conclusions: (1) data source triangulation (using multiple data sources and informants); and 2) investigator (using more than 1 person to collect, analyze, and interpret data).

## Results

### Characteristics of Caregivers and Family Physicians

Participant characteristics are presented in Tables 1 and 2.

**Table 1:** Participant characteristics: caregivers.

Participant ID	Gender	Relationship to patient
C-001	Female	Mother
C-002	Female	Mother
C-003	Female	Mother
C-004	Female	Mother
C-005	Female	Mother
C-006	Female	Mother
C-007	Female	Mother
C-008	Female	Mother
C-009	Female	Mother
C-010	Female	Mother
C-011	Female	Mother
C-012	Female	Mother
C-013	Female	Mother
C-014	Female	Mother
C-015	Female	Mother
C-016	Female	Mother
C-017	Female	Mother
C-018	Female	Mother
C-019	Female	Mother
C-020	Male	Brother-in-law
C-021	Female	Mother
C-022	Female	Mother
C-023	Female	Mother
C-024	Female	Mother

**Table 2:** Participant characteristics: family physicians.

Participant ID	Gender	Years in practice
FP-001	Male	6
FP-002	Male	12
FP-003	Female	1
FP-004	Male	3
FP-005	Male	8
FP-006	Male	10
FP-007	Female	5
FP-008	Male	20
FP-009	Male	19
FP-010	Male	10
FP-011	Male	6
FP-012	Male	24

### Caregivers’ and Family Physicians’ Perception of Needs

**Figure 1:**
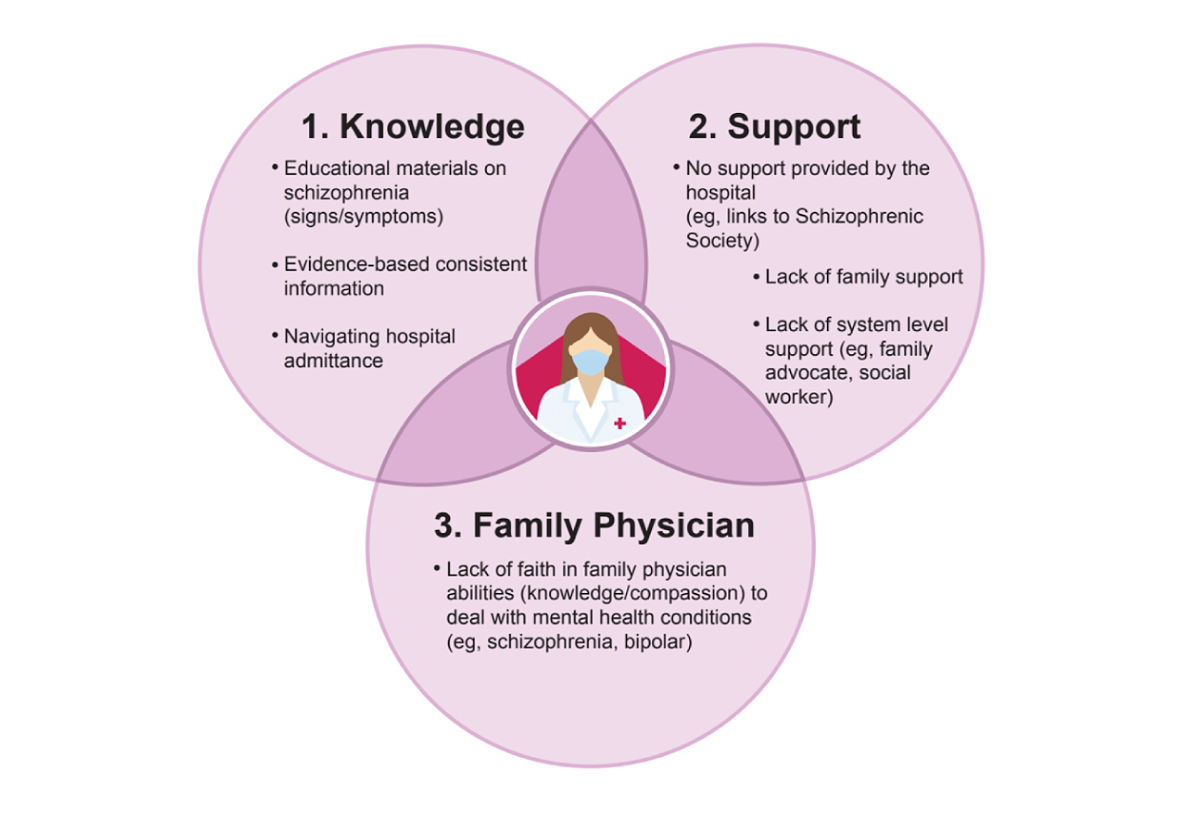
Caregiver perceptions of needs.

**Figure 2:**
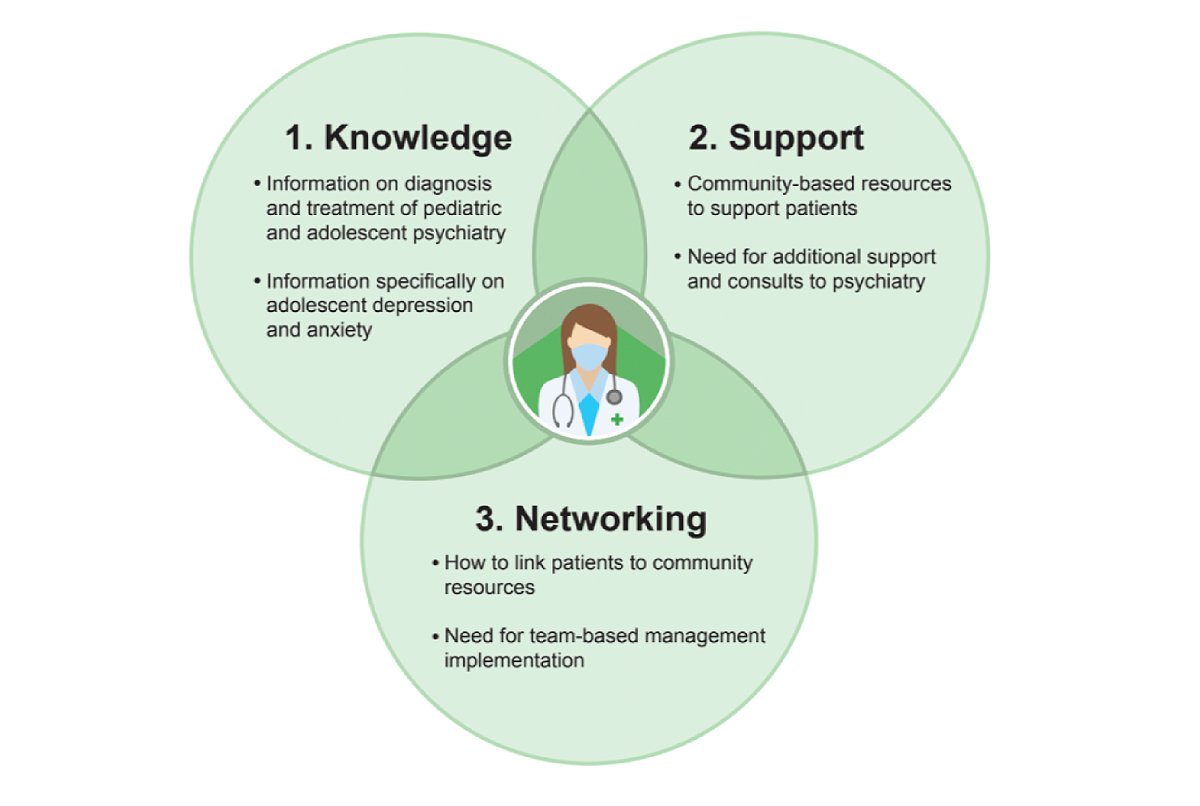
Family physician perceptions of needs.

Based on caregiver focus group data and family physician interview data, Figures 1 and 2 present the three main themes that pertained to each stakeholder group’s perceived needs. Table 3 presents each group’s preferred format of education materials, and Table 4 presents each group’s preferred method of delivery of educational materials. Prototypical qualitative quotes are provided to illuminate the themes. As shown in Figure 1, when asked about their overall needs in caring for their loved ones who suffer mental health conditions, three interrelated themes were provided from caregiver interviews: (1) the need for more knowledge, which included educational materials on the signs and symptoms of schizophrenia, evidence-based and consistent information on schizophrenia and bipolar conditions, and how to navigate hospital admissions; (2) the need for more support, which included support at the family, local hospital, and system-wide supports; and (3) wanting more support from family physicians for mental health medication management.

**Table 3:** The preferred format of education materials by stakeholder groups.

Caregiver	Family physician
Networking opportunities (in person or virtual)	Self-learning modules
Availability of support staff (eg, social workers, family advocate)	Lists of referral options
Peer support groups (face-to-face)	Support materials (eg, decision trees “if- then”)
Education sessions (eg, training sessions, lectures, seminars)	Hard copy resources (eg, Hamilton Depression Scale, Psychiatry checklists, PHQ9, CanMat Guidelines—pocket guide for depression)
System-level supports (eg, On Track, Mobile Crisis Unit, ACT [Assertive Community Treatment])	Social networks for referral or information

**Table 4:** The preferred method of delivery by stakeholder groups.

Caregiver	Family physician
Easy-to-use materials (eg, copy the “common signs and symptoms of stroke” for schizophrenia)	Documents (eg, decision trees, lists of medications with associated side effects, pocket style guides Websites such as eMentalHealth, Centre for Addiction and Mental Health, Canadian Mental Health Association, and other major mental health organizations Ottawa Depression Algorithm
Education sessions (eg, [Name] Hospital has a 2-day information session for families	Self-learning modules
Web-based peer social networking	Professional online networking or referral services Project ECHO (Extension for Community Healthcare Outcomes) Collaborative mental health networks of the Ontario College of Family Physicians

Many caregivers explained their frustrations with the process of actually having their loved ones admitted to the hospital. The following quotes are prototypical statements from participant caregivers:

#### Knowledge

My biggest complaint, most of you have heard me talk about it but what do you have to do to get someone admitted. Do you have to kill someone to get into [psychiatric hospital name]?

#### Support

They [hospitals] should also have the knowledge to tell us about the supports, about On Track, about ACT (Assertive Community Treatment), some things are out there that could help us all. I’ve never been to anything except when I met [name], by chance, and found out about [name] and the sessions put on by the Schizophrenia Society of Ontario.

I am glad that I am here for my son because the system, what is out there for you, huh, we’ve never been able to get a caseworker. I am his caseworker. I got lucky and was connected to Dr [name] at the early episode clinic. I am concerned about what will happen when I am older or not around. What will happen then? There are no supports!

#### Family Physician

Interviewer: What was your first point of contact with the system?

Caregiver: We took our son to our General Practitioner (GP), we talked to him. Our son was an honour student and suddenly he started not being interested in school and sleeping all the time. This is funny…now…but I remember the GP telling me not to worry and he said, “well at least it’s not something like schizophrenia” which is eventually what the diagnosis was. He recommended us to a psychologist.

Interviewer: How many times did you take your son back to the GP?

Caregiver: About three more times…until he had a major break then we took him to the emergency at the Royal [Ottawa]. That’s when there was a real emergency here… I probably saw the doctor with [son’s name] about seven times in six months. His symptoms weren’t obviously psychiatric. He was having vision anomalies but his vision was fine. Finally, he did have a complete break and I couldn’t get him into CHEO (Children’s Hospital of Eastern Ontario) and he was completely psychotic by then. I had to go to work one day so I asked my Mom. I said, ‘listen he hasn’t slept in a couple of days and that is not healthy, could you take him to the doctor [our GP} to get him a sedative while we wait for his assessment…he had to sleep. I came back from work that evening and he got a diet! He’s got a diet. I said to my Mom, ‘what did you tell him?”. They said that he was looking a little thin. Well, yeah, he’s not eating because he thinks the food is poisoning him so now he has a diet. [Son’s name] says, ‘the diet is the answer. If I eat this and at 10 am I eat a muffin I will be fine…a diet is an answer…it’s all good now, Mom.’. Yeah, that was the help we got at the GP’s office.

Family physicians’ needs were not all that different from those of caregivers. Figure 2 illustrates the three interrelated themes from the family physician interviews: (1) the need for more knowledge, particularly about pediatric and adolescent psychiatric conditions; (2) the need for additional support from the community and for psychiatry consults; and (3) the need for networking in terms of linking patients to community resources and the implementation of team-based management options.

#### Support

I recently had a 39-year-old female with the following diagnoses join my practice: post-traumatic stress disorder with psychotic hallucinations, major depression with psychotic features, schizophrenia and 2 weeks later BAD. On Seroquel 200 mg HS, Olanzapine 10 mg HS and the new addition of Aripiprazole 10 mg as per emergency psychiatrist with decreasing doses and eventual discontinuation of Quetiapine. Patient is engaging in high-risk behaviour. I am unable to find a psychiatrist for follow-up. What is my next step?

#### Knowledge

It would be helpful to have a list of community resources or even self-help resources. People who are mentally unwell are not going to access eHealth!

Honestly, these people [mentally ill] are the bane of my existence… this is not why I went into medicine.

#### Networking

I joined this practice a little over a year ago. This is a rural community, and I am not from here…I honestly am not so sure who or where I can refer my patients. Most of them end up going to emerg.

Caregivers and family physicians were asked about their preferred format of educational materials or knowledge. The 2 stakeholder groups were more similar than different in the preferred formats for materials (Table 3), though caregivers tend to prefer more face-to-face interaction over virtual formats. Table 3 illustrates the preferred methods of educational material delivery by stakeholder group. Caregivers and family physicians both reported preferences for both hard copy materials as well as online learning modes.

Table 4 illustrates the preferred methods of educational material delivery by stakeholder groups. Caregivers and family physicians both reported preferences for both hard copy materials as well as online learning modes.

## Discussion

### Principal Results

The objective of this needs assessment was to better understand the education and information needs of (1) caregivers of patients with mental health needs in primary care, with a focus on early recognition, diagnosis, and treatment of schizophrenia, bipolar mood disorder, and depression; and (2) family physicians’ perceptions of barriers to care. In this study, caregivers and family physician needs regarding caring for people with mental health conditions were generally similar. That is, both groups sought information such as related disease-specific symptoms and treatment options, and access to system-level psychiatric oversight. In terms of modes of delivery and educational formats for delivery, caregivers and family physicians were decisive in their preferred approaches.

The majority of caregivers had preferences for face-to-face delivery but were open to easy-to-use materials such as those developed for other diseases (eg, stroke). In keeping with adult learning principles, general practitioners desired self-learning modules with a focus on a decision tree type list of medications and side effects [9].

Most Canadians who receive mental health care do so in primary care settings, where collaborative care models have been shown to improve access to mental health care, individual and population outcomes, and cost-effective care [6]. Collaborative care involves providers from different specialties, disciplines, or sectors working together to offer complementary services and mutual support to ensure that patients receive the most appropriate service from the most appropriate provider in the most suitable location, as quickly as necessary, and with minimal obstacles [10].

The most empirically supported models of care are based on Wagner’s chronic care model, yet they are typically implemented without evaluation. This is a crucial problem because the poor implementation of collaborative care yields worse experiences and outcomes of care [6]. Patient engagement becomes central to ongoing research, design, and implementation of collaborative care. Working in partnership with patients and their caregivers can provide unique insights into their needs and how programs should be designed, evaluated, and improved.

Caregivers have asked for improvement-oriented interventions such as educational sessions. This need is not a new one and has been available in the psychiatry literature since the mid-1980s [11]. Such education sessions include psychoeducational models of family therapy that include all-day survival skills workshops initially for families of schizophrenic patients. In these workshops, professionals share with families what is and is not known about the illnesses and seem to consolidate, in a multiple family setting, the connecting process they begin with each family. The format can serve as an excellent framework for similar psychoeducational workshops with families of patients with other mental illnesses. Though the workshops have been found to have positive outcomes [12], they have not gained a lot of traction in practice likely due to a lack of consistent funding [13]. Currently, there are existing initiatives that attempt to fill these needs, which will be discussed in the following section.

### Initiatives and Resources

#### Need for Educational Materials

1. eMentalHealth [14], which is a comprehensive mental health website that provides information about mental health. It is also a resource directory on where to find help. eMentalHealth is targeted to both the general public and primary care providers, and it has been positively evaluated [15].
2. The Ottawa Depression Algorithm [16], which is a website designed to make it easy for primary care providers to diagnose and manage depression in an online decision tree format.

#### Need for Networking or Mentorship Opportunities

1. The Collaborative Mental Health Network is an initiative of the Ontario College of Family Physicians and provides mentorship support for family physicians to support patients with mental health needs.
2. Project ECHO (Extension for Community Healthcare Outcomes), which is a best practice in providing distance education about a variety of topics. In the province of Ontario, Project ECHO is funded by the Ministry of Health and provides networking and teaching in topics such as mental health.
3. There is a Project ECHO for adult mental health [17] as well as for child and youth mental health [18].

The information from this study will help inform existing initiatives and identify gaps where improvements can be made.

### Strengths and Limitations

The strengths of our study include the richness of the data obtained through firsthand accounts from key stakeholder groups. Caregivers spoke in detail about the difficulties they encountered accessing care for their loved one. In turn, family physicians voiced their concerns for a system with a lack of resources and a general dearth of information regarding psychiatric treatment options.

The limitations of our study include the small sample size of participants, which limits the generalizability of our findings. Despite the efforts to include male participants in the caregiver focus groups, our sample was predominantly female. Further, the purposive and snowball sampling strategy may create a self-selection bias in our data; as the focus groups included individuals who were willing to participate voluntarily, the results may be positively biased in favor of the study’s intent.

### Conclusion

This needs assessment demonstrated that caregivers’ and family physicians’ needs about the care and medical management of individuals with mental health conditions may not be so different. Collaboratively designed and carefully developed educational materials, delivered in preferred formats, are an important step toward effective collaborative care. As family physicians and primary care teams are better equipped to manage patients with mental health conditions, and as caregivers are better informed and supported, our hope is that “first encounters” at the primary care setting can be skillfully managed and that care can be better executed over the longer term.

## Abbreviations

ACT: Assertive Community Treatment
CHEO: Children’s Hospital of Eastern Ontario
ECHO: Extension for Community Healthcare Outcomes
GP: general practitioner
